# Added value of tumor–stroma ratio to postsurgery circulating tumor DNA and pTN stage in risk stratification of patients with stage III colon cancer treated with adjuvant chemotherapy

**DOI:** 10.1016/j.esmoop.2025.105935

**Published:** 2026-01-02

**Authors:** I.A. Franken, F. Heilijgers, M.-C.E. Bakker, C. Rubio-Alarcón, F.H. van der Baan, M. Lemmens, P. Delis-van Diemen, M. Sausen, G.A. Meijer, M. Koopman, G.R. Vink, R.J.A. Fijneman, W.E. Mesker, J.M.L. Roodhart

**Affiliations:** 1 University Medical Center Utrecht, Department of Medical Oncology, Utrecht University, Utrecht, The Netherlands; 2 Leiden University Medical Center, Department of Surgery, Leiden, The Netherlands; 3 The Netherlands Cancer Institute, Department of Pathology, Amsterdam, The Netherlands; 4 Labcorp, Baltimore, USA; 5 Department of Research and Development, Netherlands Comprehensive Cancer Organisation, Utrecht, The Netherlands

**Keywords:** colon cancer, adjuvant chemotherapy, circulating tumor DNA, tumor–stroma ratio, biomarker

## Abstract

**Background:**

Patients with stage III colon cancer (CC) are routinely treated with resection followed by adjuvant chemotherapy (ACT). Half of patients are cured by surgery alone and overtreated with ACT, yet another ∼30% experience disease recurrence. Upfront risk stratification requires biomarkers beyond the conventional pathological stage (pTN). Detection of postsurgery circulating tumor DNA (ctDNA) is indicative of minimal residual disease and prognostic of recurrence. However, its negative predictive value is insufficient to guide treatment de-escalation. This study aimed to investigate whether adding tumor–stroma ratio (TSR) can improve patient risk stratification.

**Patients and methods:**

This study included 206 patients from PLCRC-PROVENC3 based on radical resection of stage III CC followed by ACT. Postsurgery ctDNA status was determined using Labcorp® Plasma Detect™. On a hematoxylin–eosin resection section, the TSR was scored by trained observers and dichotomized as stroma-low (≤50% stroma) versus stroma-high (>50%). The primary outcome was time to recurrence in univariable and multivariable Cox analyses to inform risk-group stratification, reporting hazard ratios (HR) and recurrence risks (RR).

**Results:**

Postsurgery ctDNA was the strongest predictor [*n* = 26, 3-year RR 65.4% (95% CI 41.3% to 79.6%) versus 16.8% (95% CI 11.0% to 22.3%); HR 6.1 (95% CI 3.4-10.8)], followed by pT4/pN2 [*n* = 80, HR 3.0 (95% CI 1.7-5.2)] and a stroma-high tumor [*n* = 88, HR 3.0 (95% CI 1.7-5.2)]. Within the ctDNA-negative subgroup (*n* = 180), we identified a low-risk group [pT1-3N1 and stroma-low; *n* = 72, 3-year RR 2.9% (95% CI 0% to 6.7%)], intermediate-risk group [either pT4/N2 or stroma-high; *n* = 68, 3-year RR 17.2% (95% CI 7.4% to 26.0%), HR 8.2 (95% CI 1.9-36.2)], and high-risk group [pT4/N2 and stroma-high; *n* = 40, 3-year RR 40.3% (95% CI 22.9% to 53.9%), HR 21.5 (95% CI 5.0-92.3)].

**Conclusions:**

Adding TSR to ctDNA and pTN stage improved risk stratification of stage III CC patients receiving ACT. One-third of the patients had none of the biomarkers and could be considered for de-escalation based on their very low RR. In addition to ctDNA-positive patients, ctDNA-negative patients with a pT4/N2 stroma-high tumor may require treatment escalation to reduce their high RR.

## Highlights

• Tumor–stroma ratio and ctDNA have complementary value in stratifying RR after ACT in stage III CC patients.
• Postsurgery ctDNA-positive patients have the highest 3-year RR of 65%.
• Within the ctDNA-negative group, pathological T4/N2 stage and a stroma-high tumor identifies a high-risk group with 40% RR.
• Based on ctDNA-negativity, pT1-3N1 and stroma-low, one-third of the patients are identified as very low risk with <3% RR.

## Introduction

Colon cancer (CC) is the third most prevalent cancer globally and the second leading cause of cancer-related mortality. Patients with stage III CC are recommended surgical resection followed by adjuvant chemotherapy (ACT).^1^ Worldwide, the standard ACT consists of a fluoropyrimidine combined with oxaliplatin for patients deemed fit for treatment. In the Netherlands, the preferred regimen is 3 months of capecitabine with oxaliplatin (CAPOX).^2^

Of all stage III CC patients, 50% would have been cured by surgery only and these patients are currently overtreated with ACT.^3^ On the other hand, 30% of the patients experience recurrence of disease despite ACT and are in need of improved adjuvant treatment. A better identification of these respective low- and high-risk groups is warranted to enable individualized ACT recommendations. This highlights the need for biomarkers to improve risk stratification, beyond the current definition of low-risk (pT1-3N1) and high-risk (pT4 and/or N2) stage III CC based on pathological TNM (tumor–node–metastasis) stage.^4^

Circulating tumor DNA (ctDNA) allows for the detection and monitoring of minimal residual disease (MRD) after resection of the tumor. Several studies demonstrated that ctDNA detection after surgery has a strong prognostic value for an increased risk of recurrence (RR) in stage III CC patients treated with ACT.^5^, ^6^, ^7^, ^8^, ^9^ However, in using negative ctDNA results to guide clinical decisions toward de-escalation of treatment, false-negative ctDNA results remain a concern. To improve negative predictive value, a combination of ctDNA with other biomarkers is needed.^10^^,^^11^

In particular, characteristics of the resected tumor tissue, involving both the tumor compartment^12^ and/or the tumor microenvironment (TME),^13^ hold potential to serve as additional prognostic biomarkers. A high level of stroma is associated with tumor progression, metastasis, and resistance to therapy.^14^, ^15^, ^16^ The tumor–stroma ratio (TSR) quantifies the percentage of stroma in the resected tumor, based on a diagnostic histopathology slide.^17^^,^^18^ Recent findings from the UNITED study validated the TSR as an independent prognostic factor in stage II-III CC, also in the subgroup treated with ACT.^19^

The objective of this study was to investigate the added value of TSR to postsurgery ctDNA and pTN stage in risk stratification of stage III colon cancer. We hypothesized that the combination of pT4/N2 and stroma-high enables the identification of patients at high RR despite no detected ctDNA after surgery, while absence of all these risk factors helps define a very-low-risk group.

## Material and methods

### Study design and participants

Patients provided written informed consent in the Prospective Dutch Colorectal Cancer cohort (PLCRC; METC 12-510, NCT02070146). The PLCRC is a nationwide study that provides the infrastructure for collection of clinical data, blood, and tissue.^20^ For the observational PLCRC substudy PROVENC3,^7^ we selected 206 patients who were diagnosed with pathological stage III CC between 2016 and 2021 and treated with ACT (CAPOX or capecitabine). The pathological tumor and node stage was based on the American Joint Committee on Cancer (AJCC) TNM classification^21^ valid at the time of diagnosis. Exclusion criteria were incomplete resection (R1 or R2, *n* = 3), neoadjuvant treatment, or prior malignancy in the past 5 years. Subgroup analyses were carried out based on microsatellite stability (MSS, *n* = 174) and chemotherapy regimen (CAPOX, *n* = 195). Clinical data were collected by trained data managers at the Netherlands Cancer Registry (NCR).

### ctDNA detection

In PLCRC-PROVENC3, a postsurgery blood sample was collected at least 4 days after surgery and before initiation of ACT.^7^ ctDNA status was determined using Labcorp® Plasma Detect™, a tumor tissue-informed approach involving integrated whole genome sequencing analyses of plasma cell-free DNA (30×), germline DNA (40×), and tumor tissue DNA (80×).^4^ To this end, formalin-fixed paraffin-embedded (FFPE) tumor blocks were obtained through the Dutch Nationwide Pathology Databank (PALGA).^22^ Patients and treating physicians remained blinded for the ctDNA result.

### TSR scoring

The tumor tissue blocks used for DNA isolation were also used to generate a 4 μm slide, which was stained with hematoxylin and eosin (H&E) and digitized (NanoZoomerXR Hamamatsu, 40×). Two trained observers (FH and WM), who were unaware of the patient outcomes and ctDNA results, scored the TSR according to the previously described method.^18^ At low magnification, the most invasive tumor area with the highest representation of stroma was selected on each slide. Subsequently, at 10× objective (100× magnification), stromal percentage was scored at 10% increments. Necrosis, mucin, vascular structures, glandular lumen, or muscle tissue were excluded from the scored area. Tumors were categorized as stroma-low (≤50% stroma) or stroma-high (>50% stroma) (examples in Supplementary Figure S1A, available at https://doi.org/10.1016/j.esmoop.2025.105935), using the predefined cut-off value with most discriminative power.^15^ In cases with no agreement between the independent observers, consensus was reached through discussion. For most cases, one diagnostic slide was available. In the few instances with multiple slides, the slide with the highest TSR was used as previously validated.^16^

### Statistical analysis

Proportions of discordant classifications between ctDNA and TSR were estimated, along with their 95% confidence intervals (CIs), using the Wilson method. Biomarker-based subgroups were compared based on categorical and continuous variables, using the χ^2^ test and independent samples *t*-test, respectively. Median follow-up from resection was reported with interquartile range (IQR). The primary endpoint was time to recurrence (TTR), defined as the first incidence of locoregional or distant recurrence and censored at last date of standard clinical follow-up. The secondary endpoint was overall survival (OS), derived from the national municipal population registry in January 2025. TTR and OS were assessed in univariable log-rank and multivariable Cox proportional hazards models, after checking that assumptions were not violated visibly or based on Schoenfeld residuals. Per biomarker or risk group, the hazard ratio (HR) and 3-year RR and 5-year OS rate were reported with 95% CIs. Statistical analyses were carried out using R studio (v4.2.2). Two-sided analyses with *P* values <0.05 were considered statistically significant.

## Results

### Description of the cohort and the biomarkers

A total of 206 patients with radical resection and adjuvant CAPOX (95%) or capecitabine (5%) were included in the analysis. Postsurgery blood samples were available at a median of 13 days after surgery (IQR 4-20 days) and ctDNA was detected in 26 patients (12.6%). The TSR was scored with a Cohen’s kappa of 0.86, indicating a strong interobserver agreement between both observers. The TSR was classified as stroma-high in 88 out of 206 patients (42.7%). When stratified on ctDNA status, 42.3% (95% CI 25.5% to 61.1%) of ctDNA-positive patients were stroma-high and 57.2% (95% CI 49.9% to 64.2%) of ctDNA-negative patients were stroma-low (*P* = 1). This resulted in a low overall concordance between ctDNA and TSR of 55.3% [95% CI 48.5% to 62.0%) (Supplementary Figure S1B, available at https://doi.org/10.1016/j.esmoop.2025.105935). Pathological T4 and/or N2 stage was observed in 38.8% of patients and was correlated with a stroma-high tumor (*P* = 0.001) but not with ctDNA detection (*P* = 0.674). Other patient and tumor characteristics did not differ significantly across ctDNA and TSR status (Table 1).

**Table 1:** Patient and tumor characteristics in the overall cohort and per biomarker

	Overall, *n* (%)	ctDNA-negative		ctDNA-positive	
		Stroma-low, *n* (%)	Stroma-high, *n* (%)	Stroma-low, *n* (%)	Stroma-high, *n* (%)
	(*n* = 206)	(*n* = 103)	(*n* = 77)	(*n* = 15)	(*n* = 11)
Age, years, mean (standard deviation)	63.1 (9.3)	62.8 (8.8)	62.6 (9.7)	63.9 (11.0)	69.3 (6.2)
Sex					
Female	96 (46.6)	56 (54.4)	32 (41.6)	5 (33.3)	3 (27.3)
Male	110 (53.4)	47 (45.6)	45 (58.4)	10 (66.7)	8 (72.7)
pTN stage					
T1-3N1	126 (61.2)	72 (69.9)	37 (48.1)	12 (80.0)	5 (45.5)
T4/N2	80 (38.8)	31 (30.1)	40 (51.9)	3 (20.0)	6 (54.5)
Tumor sidedness					
Left	117 (56.8)	59 (57.3)	46 (59.7)	7 (46.7)	5 (45.5)
Right	89 (43.2)	44 (42.7)	31 (40.3)	8 (53.3)	6 (54.5)
Microsatellite stability					
MSS	174 (84.5)	84 (81.6)	66 (85.7)	13 (86.7)	11 (100)
MSI	32 (15.5)	19 (18.4)	11 (14.3)	2 (13.3)	0 (0)
Lymphatic and/or angioinvasion					
No	91 (46.9)	47 (51.1)	33 (43.4)	7 (46.7)	4 (36.4)
Yes	103 (53.1)	45 (48.9)	43 (56.6)	8 (53.3)	7 (63.6)
Missing	12	11	1	0	0
Histology					
Adenocarcinoma	182 (88.3)	93 (90.3)	65 (84.4)	13 (86.7)	11 (100)
Mucinous	18 (8.7)	8 (7.8)	9 (11.7)	1 (6.7)	0 (0)
Other (Medullary/signet ring cell)	6 (3.0)	2 (1.9)	3 (3.9)	1 (6.7)	0 (0)
Differentiation					
Moderate	173 (85.2)	88 (86.2)	63 (84.0)	13 (86.7)	9 (81.8)
Poor	30 (14.8)	14 (13.7)	12 (16.0)	2 (13.3)	2 (18.2)
Missing	3	1	2	0	0
Site of recurrence	*n* = 54	*n* = 8	*n* = 27	*n* = 10	*n* = 9
Locoregional	4 (7.4)	0 (0)	3 (11.1)	1 (10.0)	0 (0)
Liver only	21 (38.9)	3 (37.5)	6 (22.2)	6 (60.0)	6 (66.7)
Lung only	7 (13.0)	1 (12.5)	5 (18.5)	1 (10.0)	0 (0)
Lymph only	1 (1.9)	0 (0)	1 (3.7)	0 (0)	0 (0)
Peritoneum only	10 (18.5)	1 (12.5)	6 (22.2)	1 (10.0)	2 (22.2)
Multiple sites	7 (13.0)	1 (12.5)	4 (14.8)	1 (10.0)	1 (11.1)
Other	4 (7.4)	2 (25.0)	2 (7.4)	0 (0)	0 (0)

ctDNA, circulating tumor DNA; MSS/I, microsatellite stable/instable; pTN, pathological T and N stage.

### Independent prognostic value of postsurgery ctDNA, pTN stage, and TSR

Firstly, we explored the independent prognostic value of patient and tumor characteristics for recurrence of disease (Table 2). Of all 206 patients, 54 (26.3%) patients experienced a recurrence [median TTR 13.4 months (IQR 8.3-21.7 months)] and 152 remained recurrence-free [median follow-up 43.9 months (IQR 37.6-59.6 months)]. Postsurgery ctDNA-positivity was the strongest prognostic marker, identifying a small group of patients at very high RR [*n* = 26 (12.6%), 3-year RR 65.4% (95% CI 41.3% to 79.6%)] compared with ctDNA-negative patients [*n* = 180, 3-year RR 16.8% (95% CI 11.0% to 22.3%); HR 6.1, 95% CI 3.4-10.8, *P* < 0.001] (Supplementary Figure S1C, available at https://doi.org/10.1016/j.esmoop.2025.105935). Patients with a pT4 and/or N2 tumor [*n* = 80 (38.8%), 3-year RR 38.7% (95% CI 26.7% to 48.8%)] were at higher risk than patients with a pT1-3N1 tumor [*n* = 126, 3-year RR 13.1% (95% CI 6.9% to 18.9%); HR 3.0, 95% CI 1.7-5.2, *P* < 0.001] (Supplementary Figure S1D, available at https://doi.org/10.1016/j.esmoop.2025.105935). As for the TSR, the patients with a stroma-high tumor had an increased risk [*n* = 88 (42.7%), 3-year RR 33.1% (95% CI 22.5% to 42.3%)] compared with patients with a stroma-low tumor [*n* = 118, 15.2% (95% CI 8.3% to 21.7%); HR 3.0, 95% CI 1.7-5.2, *P* < 0.001] (Supplementary Figure S1E, available at https://doi.org/10.1016/j.esmoop.2025.105935).

**Table 2:** Postsurgery ctDNA, pathological stage, and TSR in univariable and multivariable models for recurrence

Variable	Level	*n*	Univariable			Multivariable		
			HR	95% CI	*P* value	HR	95% CI	*P* value
ctDNA	Detected	28	6.1	(3.5-10.8)	<0.001	8.3	(4.6-15.0)	<0.001
pTN stage	pT4/N2	82	3.0	(1.7-5.2)	<0.001	3.1	(1.8-5.5)	<0.001
TSR	Stroma-high	88	3.0	(1.7-5.2)	<0.001	2.7	(1.5-4.7)	0.001

ctDNA, circulating tumor DNA; HR, hazard ratio; pTN, pathological stage; TSR, tumor–stroma ratio.

### Complementary prognostic value of postsurgery ctDNA, pTN stage, and TSR

Notably, within the ctDNA-negative group (*n* = 180), 35 (19.4%) patients experienced recurrence, of whom 27 (77%) were stroma-high (Table 1). Especially, recurrence of disease in peritoneum and lung is missed by postsurgery ctDNA, as published before (false-negative 60%-80%^23^^,^^24^). Of the 10 patients who developed peritoneal metastases, 7 were ctDNA-negative after surgery, of whom 6 had a stroma-high tumor. Of seven patients with lung metastases, six were ctDNA-negative of whom five had stroma-high tumors. When combined in a multivariable Cox model, the three biomarkers ctDNA (HR 8.3, 95% CI 4.6-15.0, *P* < 0.001), pTN stage (HR 3.1, 95% CI 1.8-5.5, *P* < 0.001), and TSR (HR 2.7, 95% CI 1.5-4.7, *P* = 0.001) maintained prognostic value (Table 2; Supplementary Figure S2, available at https://doi.org/10.1016/j.esmoop.2025.105935).

### Combining ctDNA, pTN stage, and TSR to stratify patients into clinically relevant risk groups

**Figure 1:**
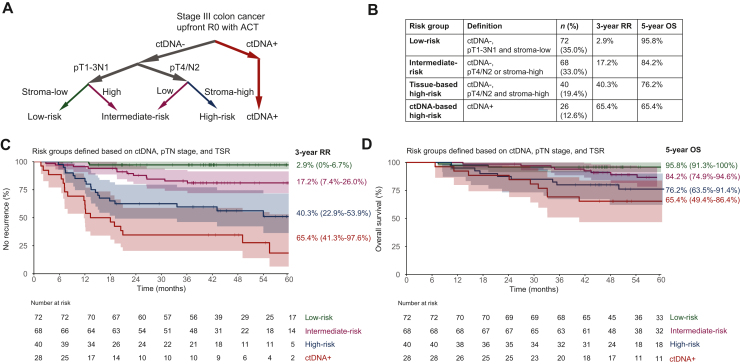
**Risk stratification system based on ctDNA, pTN stage, and TSR.** (A) Flow chart depicting the definition of the risk groups. (B) Table showing definition, number of patients and event rate per risk group. (C) 3-year RR per risk group. (D) 5-year OS per risk group. ctDNA, circulating tumor DNA; HR, hazard ratio; OS, overall survival; RR, recurrence risk; TSR, tumor–stroma ratio.

To stratify patients into clinically relevant subgroups with distinct risk, we combined postsurgery ctDNA, pTN stage, and TSR (Figure 1A and B). A low-risk group was defined as having no detectable ctDNA and having none of the tissue-based risk factors: pT1-3N1 and stroma-low. This group consisted of 35.0% of all patients (*n* = 72) and these patients had a very low RR [3-year RR 2.9% (95% CI 0% to 6.7%)] after ACT (Figure 1C). ctDNA-negative patients with only one of the other risk factors, either pT4/N2 or stroma-high, showed an intermediate risk [*n* = 68 (33.0%), 3-year RR 17.2% (95% CI 7.4% to 26.0%); HR 8.2, 95% CI 1.9-36.2, *P* = 0.005]. ctDNA-negative patients were tissue-based high-risk in case of both pT4/N2 and stroma-high [*n* = 40 (19.4%), 3-year RR 40.3% (95% CI 22.9% to 53.9%); HR 21.5, 95% CI 5.0-92.3, *P* < 0.001]. Highest risk was observed in the patients with ctDNA detection after surgery, who were therefore considered as a separate risk group [*n* = 26 (12.6%), 3-year RR 65.4% (95% CI 41.3% to 79.6%); HR 47.2, 95% CI 11.0-203.8, *P* < 0.001]. Similar to RR, OS was better in the low-risk group [5-year OS 95.8% (95% CI 91.3% to 100%)] compared with the intermediate-risk group [5-year OS 84.2% (95% CI 74.9% to 94.6%); HR 2.7, 95% CI 0.8-8.5, *P* = 0.097], the tissue-based high-risk group [5-year OS 76.2% (95% CI 63.5% to 91.4%); HR 5.8, 95% CI 1.9-18.1, *P* = 0.002], and the ctDNA-positive group [5-year OS 65.4% (95% CI 49.4% to 86.4%); HR 9.4, 95% CI 3.0-29.7, *P* < 0.001] (Figure 1D).

### The risk groups remain prognostic only for patients with MSS tumors and patients treated with CAPOX

**Figure 2:**
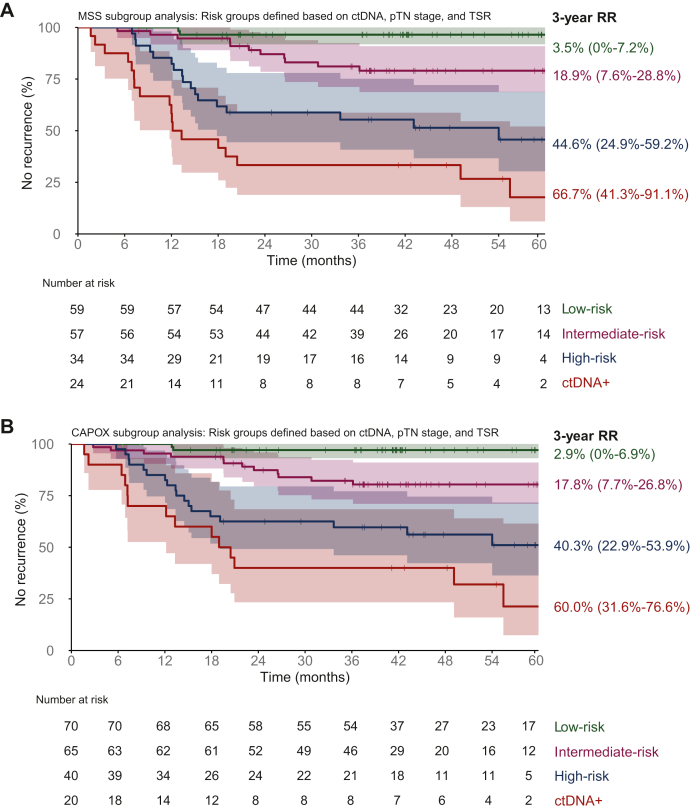
**Stratification system based on ctDNA,** pTN stage and TSR in sensitivity analysis based on (A) subgroup with MSS tumors and (B) patients treated with adjuvant CAPOX. CAPOX, capecitabine and oxaliplatin; ctDNA, circulating tumor DNA; HR, hazard ratio; MSS, microsatellite stable; RR, recurrence risk; TSR, tumor–stroma ratio.

Most patients had an MSS tumor (*n* = 174), which differ in tumor biology and treatment response from microsatellite instable (MSI) tumors and were therefore selected for a sensitivity analysis. In our cohort, MSS status was associated with higher RR than a MSI tumor [*n* = 32; HR 3.4, 95% CI 1.1-10.9, *P* = 0.039] and a nonsignificant enrichment in stroma-high tumors (44.3% versus 34.5%, *P* = 0.399). In the MSS subgroup, we confirmed the univariable and multivariable prognostic value of ctDNA, pTN stage, and TSR (Supplementary Table S1, available at https://doi.org/10.1016/j.esmoop.2025.105935). The proposed low-risk, intermediate-risk group (HR 7.1, 95% CI 1.6-31.7), tissue-based high-risk group (HR 19.4, 95% CI 4.5-83.8), and ctDNA-based high-risk group (HR 40.8, 95% CI 9.4-176.4) remained prognostic (Figure 2A). Similarly, in a sensitivity analysis focusing on only patients treated with adjuvant CAPOX (*n* = 195), excluding patients treated with capecitabine monotherapy (*n* = 11)*,* the biomarkers and proposed risk groups successfully stratified RR (Supplementary Table S2, available at https://doi.org/10.1016/j.esmoop.2025.105935; Figure 2B).

## Discussion

This study aimed to evaluate the added value of TSR to postsurgery ctDNA and pTN in stage III CC patients treated with ACT. After confirming univariable and multivariable prognostic significance of all three biomarkers, we used them to stratify patients into clinically relevant risk groups. One-third of patients were considered low risk based on ctDNA-negativity, pT1-3N1 stage, and a stroma-low tumor. ctDNA-negative patients with pT4/N2 or stroma-high were intermediate risk, and those with a pT4/N2 and stroma-high tumor were tissue-based high risk, complementing the ctDNA-based high-risk group.

Our findings support that liquid biopsy ctDNA testing should be combined with other (tissue-based) biomarkers in multimodal assessment of RR.^11^^,^^25^ Postsurgery detection of ctDNA indicates the presence of MRD and is the strongest prognostic biomarker to date in stage III CC patients treated with ACT. However, ctDNA is only detected in 11%-20% of patients and in ctDNA-negative patients the observed RR (∼20% with ACT) is too high to withhold ACT.^5^, ^6^, ^7^, ^8^, ^9^ False-negative ctDNA results have been associated with release of cell-free DNA from healthy tissue after surgery in some studies^26^ and current ctDNA assays fail to detect part of (micro)metastases, especially from low-shedding sites like the lung and peritoneum.^23^^,^^24^^,^^27^^,^^28^ Therefore, unless ctDNA assays are improved, ctDNA results are insufficient to stratify patients to safely withhold ACT, and should be combined with other biomarkers. Pathological stage reflects the locoregional tumor extent and adding pathological stage helps to delineate a lower risk ctDNA-negative subgroup based on pT1-3N1.^7^^,^^29^^,^^30^ We now show a further clinically relevant reduction in 3-year RR to <3% in the subset of patients with ctDNA-negative, pT1-3N1 stroma-low tumor.

The complementary value of the TSR is evident from the observation that recurrences in ctDNA-negative patients, particularly in the peritoneum and lungs, occurred predominantly in patients with stroma-high primary tumors. The TSR as prognostic biomarker was previously described in the UNITED study, which observed a comparable proportion of stroma-high tumors (39%) and prognostic impact (event rate 34% versus 20%, HR 1.7).^19^ These findings, together with the almost perfect agreement between observers, underline the robustness of the TSR. Moreover, the TSR can be easily integrated in the diagnostic workflow, as it can be determined during standard pathological staging on the same H&E slide of the deepest invasive front.^18^^,^^31^ Therefore, concurrent use of pathological stage and TSR may stratify tissue-based risk within a few days after surgery.

A limitation in the interpretation of the study results is that all patients were treated with ACT, confirming with the guideline. Although we identified a large subgroup with very low risk after ACT, a control group of patients without ACT is required to inform whether de-escalation of treatment would be safe. Future studies should prospectively validate the proposed risk stratification based on ctDNA, pTN stage, and TSR, preferably randomizing the low-risk patients between ACT and surgery only. This may help inform whether addition of ACT (OS >95% in our study) provides a clinically relevant benefit, defined as >5% improvement in OS.^32^^,^^33^ If the benefit from ACT does not outweigh the potential toxicity, especially neuropathy,^34^ low-risk patients may decide to withhold ACT until ctDNA becomes detectable in serial monitoring during intensive follow-up.^35^ For the high-risk patients, it remains to be investigated what escalation treatment would provide benefit. ctDNA studies show that half to two-thirds of patients with MRD are not cured by adjuvant CAPOX,^5^, ^6^, ^7^, ^8^, ^9^ and recent results suggest that this is not improved by longer CAPOX duration or escalation to FOLFOXIRI.^36^ High stroma may provide a novel target for therapy, based on our finding that stroma-high tumors exhibit a significantly worse outcome. The reduced sensitivity to standard fluoropyrimidine–oxaliplatin-based therapy of stroma-high tumors is consistent with prior findings on consensus molecular subtype 4^6^ and cancer-associated fibroblasts.^37^ This suggests that for these patients, the TME may provide a potential target for novel treatment options^38^, like agents targeting transforming growth factor β (galunisertib^39^) or fibroblast growth factor (pemigatinib^40^) or fibroblast activation protein (talabostat^41^).

In conclusion, the combination of ctDNA, pTN stage, and TSR holds potential to improve risk stratification of patients with stage III colon cancer, specifically to identify false-negative ctDNA results after surgery. The ctDNA-negative patients with a pT1-3N1 stroma-low tumor were at very low RR. These patients may currently be overtreated with ACT and may be considered for de-escalation in the future. Conversely, patients with a stroma-high pT4/N2 tumor, in addition to all ctDNA-positive patients, were at high RR despite standard ACT. They may currently be undertreated and may be selected for treatment escalation. Multimodal strategies combining ctDNA and tissue-based biomarkers may help the prediction of RR and inform personalized treatment decision making.

## Supplementary data

Supplementary Figure 2

Supy Mplementary Tables and Figures Legend
