# Supplementary material for: Added value of tumor–stroma ratio to postsurgery circulating tumor DNA and pTN stage in risk stratification of patients with stage III colon cancer treated with adjuvant chemotherapy
Source: ESMO Open. 2026 Jan 2;11(1):105935. doi: 10.1016/j.esmoop.2025.105935 (PMC12805340; doi:10.1016/j.esmoop.2025.105935)
Supplement: Supplementary Figure 1 [file mmc1.pdf]

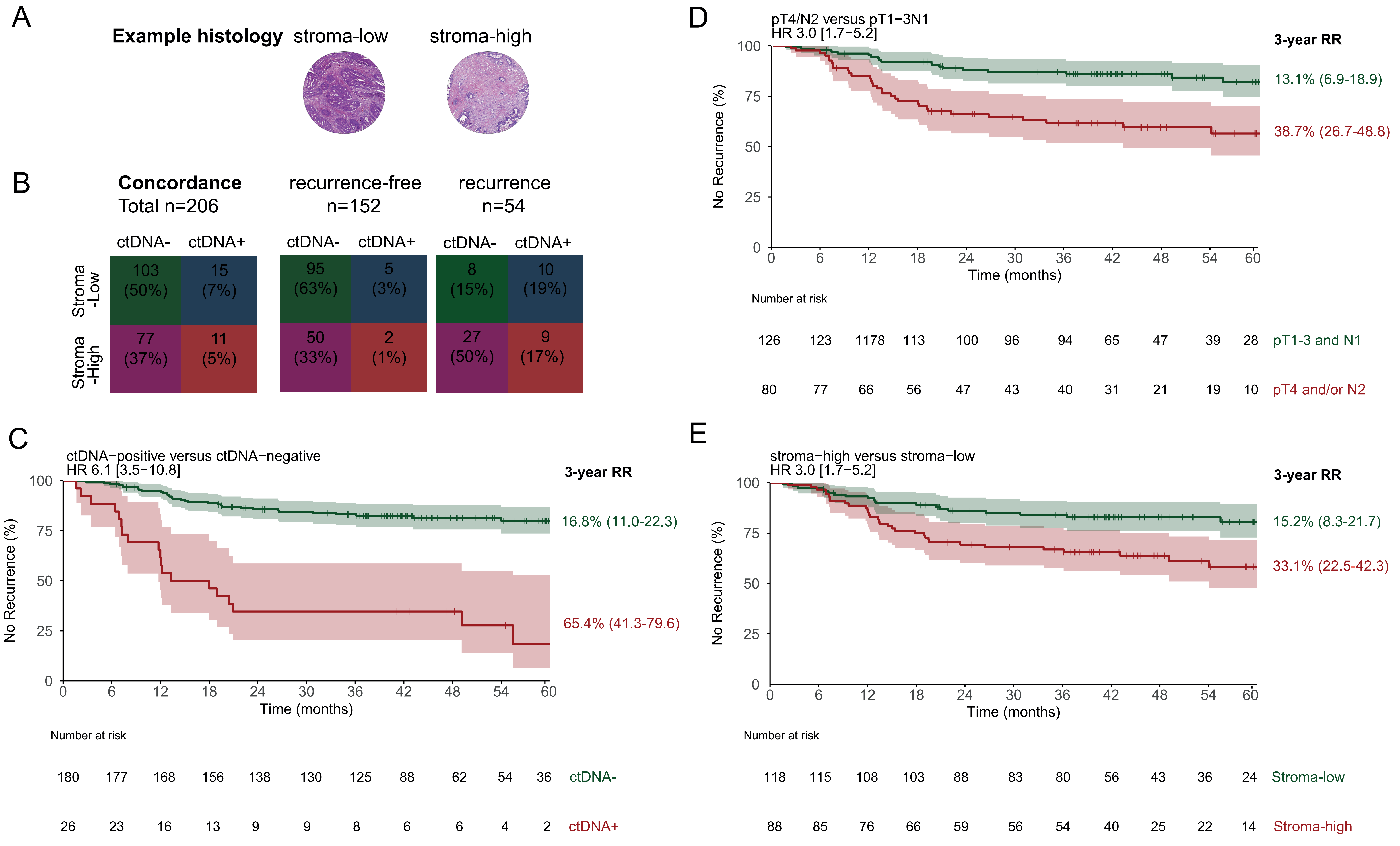
A
Example histology
stroma-low
stroma-high
B
Concordance
Total n=206
recurrence-free
n=152
recurrence
n=54
ctDNA-
ctDNA+
Stroma -Low
Stroma -High
103 (50%)
15 (7%)
77 (37%)
11 (5%)
95 (63%)
5 (3%)
50 (33%)
2 (1%)
8 (15%)
10 (19%)
27 (50%)
9 (17%)
C
ctDNA−positive versus ctDNA−negative
HR 6.1 [3.5−10.8]
3-year RR
16.8% (11.0-22.3)
65.4% (41.3-79.6)
No Recurrence (%)
Time (months)
Number at risk
180 177 168 156 138 130 125 88 62 54 36 ctDNA-
26 23 16 13 9 9 8 6 6 4 2 ctDNA+
D
pT4/N2 versus pT1−3N1
HR 3.0 [1.7−5.2]
3-year RR
13.1% (6.9-18.9)
38.7% (26.7-48.8)
No Recurrence (%)
Time (months)
Number at risk
126 123 1178 113 100 96 94 65 47 39 28 pT1-3 and N1
80 77 66 56 47 43 40 31 21 19 10 pT4 and/or N2
E
stroma−high versus stroma−low
HR 3.0 [1.7−5.2]
3-year RR
15.2% (8.3-21.7)
33.1% (22.5-42.3)
No Recurrence (%)
Time (months)
Number at risk
118 115 108 103 88 83 80 56 43 36 24 Stroma-low
88 85 76 66 59 56 54 40 25 22 14 Stroma-high
