# Supplementary material for: Added value of tumor–stroma ratio to postsurgery circulating tumor DNA and pTN stage in risk stratification of patients with stage III colon cancer treated with adjuvant chemotherapy
Source: ESMO Open. 2026 Jan 2;11(1):105935. doi: 10.1016/j.esmoop.2025.105935 (PMC12805340; doi:10.1016/j.esmoop.2025.105935)
Supplement: Supplementary Figure 2 [file mmc2.pdf]

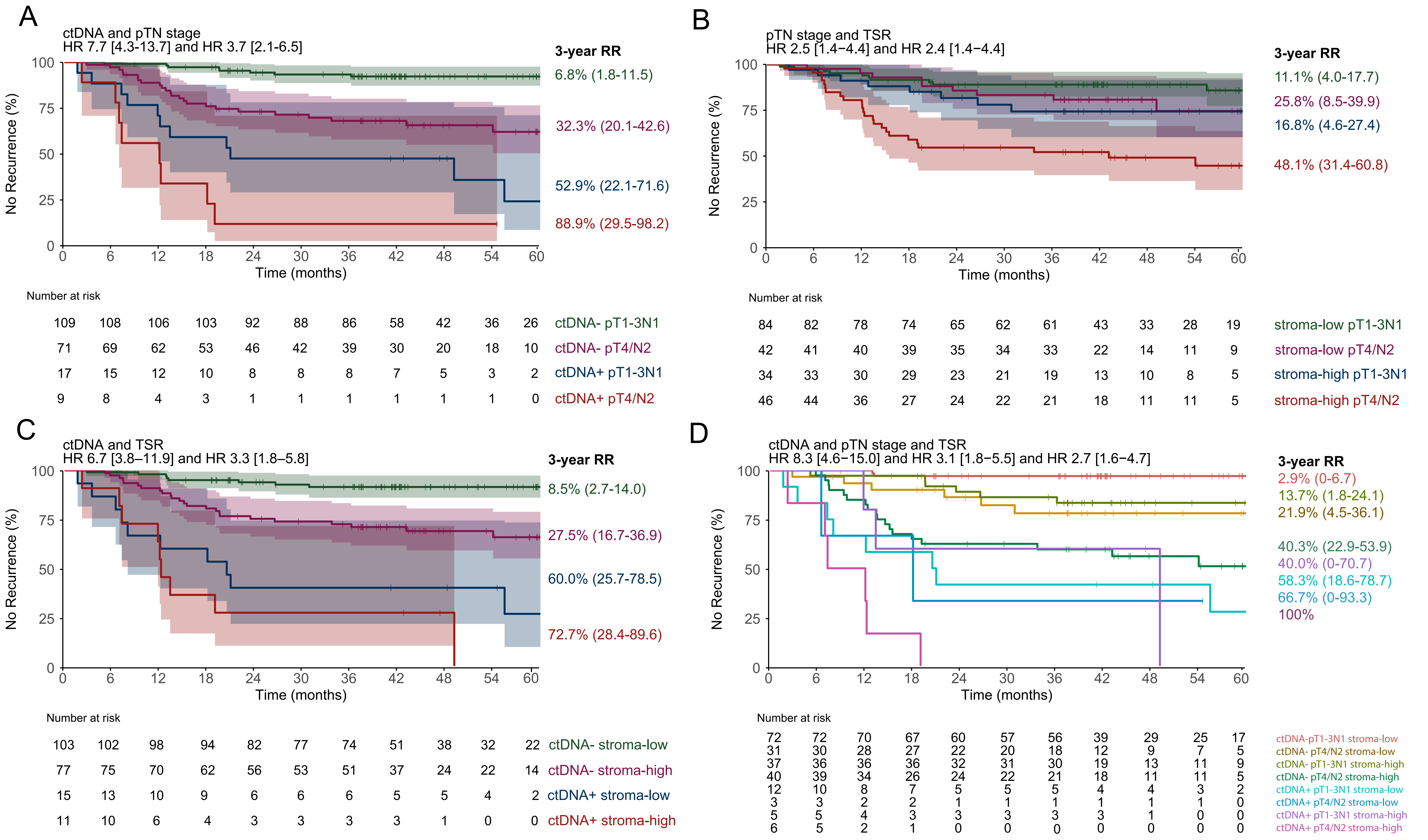
A
ctDNA and pTN stage
HR 7.7 [4.3-13.7] and HR 3.7 [2.1-6.5]
3-year RR
6.8% (1.8-11.5)
32.3% (20.1-42.6)
52.9% (22.1-71.6)
88.9% (29.5-98.2)
No Recurrence (%)
Time (months)
Number at risk
109 108 106 103 92 88 86 58 42 36 26 ctDNA- pT1-3N1
71 69 62 53 46 42 39 30 20 18 10 ctDNA- pT4/N2
17 15 12 10 8 8 8 7 5 3 2 ctDNA+ pT1-3N1
9 8 4 3 1 1 1 1 1 1 0 ctDNA+ pT4/N2
B
pTN stage and TSR
HR 2.5 [1.4−4.4] and HR 2.4 [1.4−4.4]
3-year RR
11.1% (4.0-17.7)
25.8% (8.5-39.9)
16.8% (4.6-27.4)
48.1% (31.4-60.8)
No Recurrence (%)
Time (months)
Number at risk
84 82 78 74 65 62 61 43 33 28 19 stroma-low pT1-3N1
42 41 40 39 35 34 33 22 14 11 9 stroma-low pT4/N2
34 33 30 29 23 21 19 13 10 8 5 stroma-high pT1-3N1
46 44 36 27 24 22 21 18 11 11 5 stroma-high pT4/N2
C
ctDNA and TSR
HR 6.7 [3.8−11.9] and HR 3.3 [1.8−5.8]
3-year RR
8.5% (2.7-14.0)
27.5% (16.7-36.9)
60.0% (25.7-78.5)
72.7% (28.4-89.6)
No Recurrence (%)
Time (months)
Number at risk
103 102 98 94 82 77 74 51 38 32 22 ctDNA- stroma-low
77 75 70 62 56 53 51 37 24 22 14 ctDNA- stroma-high
15 13 10 9 6 6 6 5 5 4 2 ctDNA+ stroma-low
11 10 6 4 3 3 3 3 1 0 0 ctDNA+ stroma-high
D
ctDNA and pTN stage and TSR
HR 8.3 [4.6−15.0] and HR 3.1 [1.8−5.5] and HR 2.7 [1.6−4.7]
3-year RR
2.9% (0-6.7)
13.7% (1.8-24.1)
21.9% (4.5-36.1)
40.3% (22.9-53.9)
40.0% (0-70.7)
58.3% (18.6-78.7)
66.7% (0-93.3)
100%
No Recurrence (%)
Time (months)
Number at risk
72 72 70 67 60 57 56 39 29 25 17 ctDNA-pT1-3N1 stroma-low
31 30 28 27 22 20 18 12 9 7 5 ctDNA- pT4/N2 stroma-low
37 36 36 36 32 31 30 19 13 11 9 ctDNA- pT1-3N1 stroma-high
40 39 34 26 24 22 21 18 11 11 5 ctDNA- pT4/N2 stroma-high
12 10 8 7 5 5 5 4 4 3 2 ctDNA+ pT1-3N1 stroma-low
3 3 2 2 1 1 1 1 1 1 0 ctDNA+ pT4/N2 stroma-low
5 5 4 3 3 3 3 3 1 0 0 ctDNA+ pT1-3N1 stroma-high
6 5 2 1 0 0 0 0 0 0 0 ctDNA+ pT4/N2 stroma-high
