# Supplementary material for: Added value of tumor–stroma ratio to postsurgery circulating tumor DNA and pTN stage in risk stratification of patients with stage III colon cancer treated with adjuvant chemotherapy
Source: ESMO Open. 2026 Jan 2;11(1):105935. doi: 10.1016/j.esmoop.2025.105935 (PMC12805340; doi:10.1016/j.esmoop.2025.105935)
Supplement: Supy Mplementary Tables and Figures Legend [file mmc3.docx]

**Supplementary Figure 1**: A) Example histology of TSR; B) Concordance with ctDNA, in the overall population and the subgroup with and without recurrence. Univariable time to recurrence analysis and reported recurrence rates of C) post-surgery ctDNA; D) pTN stage; E) TSR.

Abbreviations: ctDNA, circulating tumor DNA; HR, hazard ratio; pTN, pathological stage; RR, recurrence rate; TSR, tumor-stroma ratio.

**Supplementary Figure 2:** Time to recurrence analysis (no correction for other variables) of A) ctDNA and pTN stage; B) pTN stage and TSR; C) ctDNA and TSR. D) ctDNA, pTN stage and TSR.

Abbreviations: ctDNA, circulating tumor DNA; HR, hazard ratio; pTN, pathological stage; RR, recurrence risk; TSR, tumor-stroma ratio.

**Supplementary Table 1***:* post-surgery ctDNA, pathological stage and TSR in univariable and multivariable models for recurrence in sensitivity analysis based on subgroup with MSS tumors.

Abbreviations: ctDNA, circulating tumor DNA; HR, hazard ratio; pTN, pathological stage; TSR, tumor-stroma ratio.

**Supplementary Table 2:** post-surgery ctDNA, pathological stage and TSR in univariable and multivariable models for recurrence in sensitivity analysis based on subgroup treated with CAPOX.

Abbreviations: ctDNA, circulating tumor DNA; HR, hazard ratio; pTN, pathological stage; TSR, tumor-stroma ratio*.*
