# Supplementary material for: Added value of tumor–stroma ratio to postsurgery circulating tumor DNA and pTN stage in risk stratification of patients with stage III colon cancer treated with adjuvant chemotherapy
Source: ESMO Open. 2026 Jan 2;11(1):105935. doi: 10.1016/j.esmoop.2025.105935 (PMC12805340; doi:10.1016/j.esmoop.2025.105935)
Supplement: Supplementary Table 1 [file mmc4.docx]

**Supplementary Table 1***:* post-surgery ctDNA, pathological stage and TSR in univariable and multivariable models for recurrence in sensitivity analysis based on subgroup with MSS tumors.

Abbreviations: ctDNA, circulating tumor DNA; HR, hazard ratio; pTN, pathological stage; TSR, tumor-stroma ratio.

| **MSS subgroup** |  |  | **Univariable** | | | **Multivariable** | | | |  | |
| --- | --- | --- | --- | --- | --- | --- | --- | --- | --- | --- | --- |
| **Variable** | **Level** | **n** | **HR** | **95%CI** | **p-value** | **HR** | **95%CI** | **p-value** |  | |  |
| **ctDNA** | Detected | 24 | 5.8 | [3.3-10.4] | <0.001 | 7.5 | [4.1-13.6] | <0.001 |  | |  |
| **pTN stage** | pT4/N2 | 68 | 2.9 | [1.7-5.2] | <0.001 | 3.0 | [1.7-5.4] | <0.001 |  | |  |
| **TSR** | Stroma-high | 77 | 3.0 | [1.7-5.5] | <0.001 | 2.7 | [1.5-5.0] | 0.001 |  | |  |
