# Supplementary material for: Added value of tumor–stroma ratio to postsurgery circulating tumor DNA and pTN stage in risk stratification of patients with stage III colon cancer treated with adjuvant chemotherapy
Source: ESMO Open. 2026 Jan 2;11(1):105935. doi: 10.1016/j.esmoop.2025.105935 (PMC12805340; doi:10.1016/j.esmoop.2025.105935)
Supplement: Supplementary Table 2 [file mmc5.docx]

**Supplementary Table 2:** post-surgery ctDNA, pathological stage and TSR in univariable and multivariable models for recurrence in sensitivity analysis based on subgroup treated with CAPOX.

Abbreviations: ctDNA, circulating tumor DNA; HR, hazard ratio; pTN, pathological stage; TSR, tumor-stroma ratio*.*

| **CAPOX subgroup** | |  | **Univariable** | | | **Multivariable** | | | |  | |
| --- | --- | --- | --- | --- | --- | --- | --- | --- | --- | --- | --- |
| **Variable** | **Level** | **n** | **HR** | **95%CI** | **p-value** | **HR** | **95%CI** | **p-value** |  | |  |
| **ctDNA** | Detected | 20 | 5.2 | [2.8-9.6] | <0.001 | 7.7 | [4.0-14.7] | <0.001 |  | |  |
| **pTN stage** | pT4/N2 | 78 | 3.5 | [1.9-6.3] | <0.001 | 3.3 | [1.8-6.1] | <0.001 |  | |  |
| **TSR** | Stroma-high | 83 | 3.5 | [1.9-6.4] | <0.001 | 3.2 | [1.7-6.0] | <0.001 |  | |  |
